# Major Determinants of Early-Onset Coronary Artery Disease: A Descriptive Study From a Tertiary Care Hospital in Dubai, United Arab Emirates (2018-2022)

**DOI:** 10.7759/cureus.82920

**Published:** 2025-04-24

**Authors:** Dalal Obaid, Malak Dawood, Mohamad Al Hayek, Aida J Azar, Amar H Khamis, Mohamad Felo

**Affiliations:** 1 Cardiology, Mohammed Bin Rashid University of Medicine and Health Sciences (MBRU) Dubai Health, Dubai, ARE; 2 Emergency Medicine, Mediclinic Welcare Hospital, Dubai, ARE; 3 Epidemiology, Mohammed Bin Rashid University of Medicine and Health Sciences (MBRU) Dubai Health, Dubai, ARE; 4 Biostatistics, Mohammed Bin Rashid University of Medicine and Health Sciences (MBRU) Dubai Health, Dubai, ARE; 5 Cardiology, Mediclinic Welcare Hospital, Dubai, ARE

**Keywords:** cardiovascular determinants, premature coronary artery disease, south asian, united arab emirates, young adults

## Abstract

Background: Premature coronary artery disease (CAD) is becoming increasingly prevalent among young adults worldwide, including in the United Arab Emirates (UAE). This rising trend poses a significant public health concern, increasing morbidity and mortality while burdening healthcare systems. As urbanization and lifestyle changes escalate, understanding the key determinants of premature CAD is crucial. Objectives include assessing early-onset CAD patients, documenting their clinical and demographic characteristics, and identifying key contributors to disease occurrence.

Methods: A five-year descriptive study (2018-2022) was conducted using data from the catheterization laboratory of a tertiary care hospital in Dubai. Premature CAD was defined as CAD in men under 45 years and women under 55 years, confirmed by symptomatic presentation and angiographic evidence of ≥50% stenosis in coronary arteries. Cardiovascular risk factors including dyslipidemia (DLP), hypertension (HTN), gender, and body mass index (BMI) were analyzed. Continuous variables were summarized using mean (standard deviation) and categorical variables as frequencies (percentages). Chi-squared and t-tests assessed differences, with significance set at p < 0.05.

Results: Among 110 patients, 77 (70%) were men, with a mean age of 42.0 (3.6) years, significantly younger than women at 48.8 (4.4) years (p < 0.001). Most participants were Asian (70, 64%), while UAE nationals accounted for 19 (17.2%). Obesity was more prevalent among women (20, 60.6%) than men (28, 36.4%) (p = 0.063). Most patients were non-smokers (86, 78.2%), with all smokers being men (24, 31.2%) (p < 0.001). Regarding cardiovascular risk factors, 63 (57.3%) had HTN, 37 (33.6%) had diabetes mellitus (DM), and 92 (83.6%) had DLP. A significant proportion were overweight (47, 42.7%) or obese (48, 43.6%). HTN was significantly lower in men (37, 48.1%) than in women (26, 78.8%) (p = 0.003). DM was also less common in men (19, 24.7%) than in women (18, 54.5%) (p = 0.004). While DLP and family history prevalence were similar between genders, women had greater co-morbidity severity (p = 0.027). HTN was more common in overweight (18, 48.6%) and obese (17, 45.9%) men than those with normal weight (2, 5.4%) (p = 0.038). A similar trend was seen in women, with HTN being higher in overweight (7, 26.9%) and obese (18, 69.2%) individuals than those of normal weight (1, 3.8%) (p = 0.059). No significant demographic differences were observed between UAE nationals and non-nationals, though DM was lower in UAE nationals (2, 10.5%) than non-nationals (35, 38.5%) (p = 0.030). Comparing Asians (70, 63.6%) and non-Asians (40, 36.4%), Asians smoked less (10, 14.3%) than non-Asians (14, 35.0%) (p = 0.016) but had higher DM prevalence (32, 45.7%) vs. non-Asians (5, 12.5%) (p < 0.001). Asians also had greater co-morbidity severity, with 33 (47.1%) having at least three conditions compared to three (7.5%) among non-Asians (p < 0.001).

Conclusion: This study highlights key risk factors associated with premature CAD in young adults, with DLP, male gender, HTN, overweight status, and South Asian ethnicity emerging as notable associated factors. These findings underscore the need for targeted public health interventions, awareness campaigns, and lifestyle modifications to help mitigate the burden of premature CAD and improve long-term cardiovascular outcomes in the UAE.

## Introduction

Cardiovascular diseases (CVDs) are a group of disorders affecting the heart and blood vessels and are the leading cause of death globally [1]. Coronary artery disease (CAD) is the most prevalent type of CVD [2]. It is caused by plaque buildup in the coronary arteries' walls, which supply blood to the heart and other parts of the body. Plaque is a fatty, waxy substance composed of cholesterol fatty deposits, cellular waste products, calcium, fibrin, and other substances in the arteries [3]. Over time, plaque buildup can lead to atherosclerosis, the progressive thickening and hardening of the arterial walls due to the buildup of fatty plaques. This can partially or completely block blood flow to the heart, potentially resulting in a heart attack [4]. Premature CAD refers to the onset of CAD in men younger than 45 years and women younger than 55 years. It is becoming increasingly frequent among the young population [5]. The 10-year age gap in diagnosis between men and women is based on research indicating that the onset of heart disease in women typically occurs about 10 years later than in men [6]. This is primarily due to the protective effects of estrogen in premenopausal women, as estrogen positively influences lipid profiles by increasing high-density lipoprotein (HDL) cholesterol and decreasing low-density lipoprotein (LDL) cholesterol. These effects help delay the development of atherosclerosis and the development of dyslipidemia (DLP), which is one of the risk factors associated with the development of CVD [7]. DLP is an unhealthy balance of fat levels in the blood, characterized by elevated LDL cholesterol levels. This can lead to plaque buildup in the arteries, which may occlude the artery, restricting blood flow and contributing to CAD development [8].

In addition to DLP, CAD has several other risk factors, including obesity, smoking, diabetes mellitus (DM), and hypertension (HTN), which the study will focus on. HTN can increase the risk of CAD by exerting excessive force on the arterial walls, causing the coronary arteries to stretch further than usual. This leads to injury to the delicate inner lining (endothelium), which promotes plaque buildup and artery narrowing [9]. Other risk factors, such as age, gender, genetics, physical activity, and smoking, play a vital role in the severity of CAD cases [10]. Research has identified significant ethnic-specific differences in cardiovascular risk factors and variations in cardiovascular mortality worldwide. Studies have shown that South Asian and African American adults are more than twice as likely as White adults to be hospitalized for heart failure [11]. In contrast, adults of East Asian and White descent tend to have a lower susceptibility to the disease and a significantly lower cardiovascular mortality rate compared to other groups [12]. Additionally, socioeconomic status (SES) is a major determinant of CAD risk. Low SES is associated with a significantly increased risk in both men and women due to the cumulative effect of multiple behavioral and psychological risk factors. Furthermore, limited access to healthcare among individuals with low SES accelerates disease progression, as timely interventions are lacking [13]. CVD complications are increasing in developing countries and represent a key challenge in the healthcare system, nationally and globally, as it is a significant cause of death in the region, contributing to 28% of deaths in the UAE [14]. Additionally, the World Health Organization (WHO) reported that CVD is the leading cause of death worldwide in developed and developing countries, accounting for 17.9 million deaths annually (31% of all deaths) [1].

As of 2019, 80% of these deaths occurred in low-income and middle-income countries. Globally, CVDs account for approximately 85% of all disability-adjusted life years (DALYs) attributed to non-communicable diseases, highlighting the urgency of identifying modifiable risk factors and implementing effective prevention strategies [15]. It has been estimated by the WHO that if current trends continue and interventions are not enhanced, global annual CVD-related deaths will increase from 17.5 million in 2012 to 22.2 million by 2030 [1].

Premature CAD presents a greater challenge than late-onset CAD due to the limited research on its risk factors and unchanged survival outcomes. A systematic review reports that over the last three decades, while late-onset CAD has seen a notable reduction in mortality rates (5% in women and 4% in men), in contrast over the last two decades, premature CAD has only shown a minimum of 0.1% [16]. These findings highlight the urgent need to prioritize research and targeted interventions for premature CAD. Addressing this gap could lead to improved preventive strategies and better outcomes, especially since the reduction in mortality rates has been disproportionately lower in premature CAD compared to late-onset CAD. This study aims to investigate the major known risk factors contributing to the development of premature CAD among young adults by assessing newly diagnosed cases in relation to common modern societal risk factors. Identifying key contributors to CAD occurrence will help raise awareness and support preventive strategies focused on modifiable risk factors.

## Materials and methods

Study design and setting

This descriptive cross-sectional study was conducted at Mediclinic Welcare (MWEL) Hospital’s catheterization laboratory (CATH LAB) in Dubai, United Arab Emirates (UAE). MWEL is a 147-bed private tertiary care hospital with comprehensive coverage of all specialties. The hospital is in the lower to middle SES of Dubai, specifically in Old Dubai, which is home to a significant proportion of labor migrants [17]. This area is characterized by more affordable rents and accessible amenities compared to the newer and more affluent districts of the city of Dubai [18]. This contextual detail was included to help interpret the representativeness and generalizability of the study population to the broader demographic landscape of Dubai.

Eligibility criteria

Patients with documented premature CAD confirmed by coronary angiography in the CATH LAB (MWEL) between January 1, 2018, and December 31, 2022, were eligible for this study. As the study looked at early onset, also known as premature CAD, the included patients were men younger than 45 years and women under 55 years. The criteria for diagnosing CAD included symptomatic patients presenting to the emergency department who underwent coronary angiography in the MWEL CATH LAB and were found to have 50% or greater stenosis in the coronary arteries. A coronary angiogram was the diagnostic procedure used in this study for CAD diagnosis, by looking at the coronary arteries for narrowing or blockages. The procedure involves inserting a small catheter through a large artery in the groin or wrist, advancing it into the heart, and placing it at the openings of the coronary arteries. A special contrast dye is injected through the catheter and into the blood vessels, allowing live X-ray imaging to capture detailed pictures to determine the location and severity of the blockages [19]. Patients were excluded from this study if they were older than 45 years for men or older than 55 years for women. Patients with “other CAD” were defined as those with previously documented stable angina, non-obstructive CAD (<50% stenosis), or a history of myocardial infarction diagnosed outside of the current admission.

Study population size

A complete coverage population was selected using the hospital’s electronic medical records system, Bayanaty, and the Health Information System (HIS) databases, which recorded the patients’ data in the hospital, covering the period January 1, 2018, to December 31, 2022. It is the hospital's electronic medical record database system from which the information was obtained. Patient selection was based on the predefined inclusion and exclusion criteria for selecting patients with premature CAD. Records with incomplete core variables (e.g., age, diagnosis, and body mass index (BMI)) were excluded. No imputation methods were used, and patients with missing key data were not included in the final analysis. As this was a descriptive, retrospective study, no formal sample size calculation was performed. All eligible cases within the five-year study window were included.

Data source

Demographic variables included age (years), BMI (kg/m²), gender, and nationality. DM was defined as a fasting blood glucose level of 126 mg/dL (7 mmol/L) or higher on two separate tests. Additionally, diagnostic tests included oral glucose tolerance tests, two-hour plasma glucose levels (mmol/L), and random plasma glucose levels (mmol/L) [20]. HTN was diagnosed with systolic blood pressure ≥ 140 mmHg and/or diastolic blood pressure ≥ 90 mmHg on two separate days [21]. DLP was defined as blood cholesterol levels > 5.2 mmol/L (200 mg/dL), triglycerides > 1.7 mmol/L (150 mg/dL), LDL cholesterol > 2.58 mmol/L (100 mg/dL), and/or HDL cholesterol < 1.03 mmol/L (40 mmol/L) [8]. The presence of co-morbidities such as DM, HTN, and DLP was determined based on existing documentation in the electronic medical records at the time of the patient’s hospital admission. The data was stored on a password-encrypted laptop, as well as in the database of the MWEL laboratory. Only the principal investigator and co-investigators had access to the data.

Statistical methods

The data was entered into a Microsoft Excel spreadsheet (Microsoft Corp., Redmond, WA, US) and then transferred to IBM Statistical Package for Social Sciences (SPSS) version 29.0 (IBM Corp., Armonk, NY, US) for further statistical analysis. The data was analyzed based on gender, age, presence of co-morbidities, and ethnicity. The ethnicities were further categorized according to the WHO, which categorizes countries into multiple demographics based on several factors, including geographical, sociopolitical, cultural, and economic factors [22,23]. The Chi-squared test was used to test the association between the categorical variables. The mean and standard deviation (SD) were calculated for continuous variables, and the Student t-test was used to test associations. Statistical significance was set at p ≤ 0.05.

Ethical approval

Ethical approval for this undergraduate medical student research study was granted by both the Mohamed Bin Rashid University of Medicine and Health Sciences (MBRU), Dubai, UAE, Institutional Review Board (Reference # MBRU IRB-2023-37), and the Mediclinic Research Ethics Committee, Dubai, UAE (Reference # MCME.CR.SR.305.MWEL.2022).

Open Science Framework registration

The protocol of this study was registered with the open-source platform, Open Science Framework (OSF), including details on the research design, hypothesis, and method of data collection (DOI: 10.17605/OSF.IO/SM2KR; link: https://osf.io/sm2kr).

## Results

A total of 1,152 patients were recruited from the CATH LAB in MWEL from January 1, 2018, to December 31, 2022. From this group, 110 patients were diagnosed with premature CAD that fit the inclusion criteria. Table 1 shows the distribution of demographic characteristics and cardiovascular risk patterns among these 110 young adults. There were significantly more men (77 (70.0%)) than women (33 (30.0%)). The mean age of men was 42.0 years (SD = 3.6), which was significantly younger than women (48.8 years (SD = 4.4)) (p < 0.001). There were more men, 18 (23.4%), than women, 2 (6.1%), in the group younger than 40. Most men were in the age group 41 to 45 years (59 (76.6%)), and most women were in the age group 45 to 55 years (26 (79.8%)). There were no men older than 46 years as per the inclusion criteria. These data confirm the inclusion criteria where men were defined as younger than 45 years and women younger than 55 years.

**Table 1:** Demographic characteristics and cardiovascular co-morbidities by gender UAE: United Arab Emirates; SD: standard deviation; BMI: body mass index ^1^Normal weight BMI (18.5-24.9), overweight BMI (25.0-29.9), and obese BMI (≥30). ^2^African: Comorian (1), Kenyan (1), Mauritian (1), Tanzanian (1), and Algerian (1); total = 5. Middle Eastern: Egyptian (7), Iranian (1), Jordanian (4), Lebanese (3), and Syrian (1); total = 16. Asian: Filipino (9), Indian (49), Nepali (1), Pakistani (5), and Sri Lankan (6); total = 70. UAE nationals (19); total = 19. ^3^Hypertension, diabetes mellitus, dyslipidemia, and family history. *p < 0.05 indicates statistical significance.

Characteristics	Male (n = 77) N (%)	Female (n = 33) N (%)	p-value
Age (years) mean (SD) (range)	42.0 (3.6) (27-45)	48.8 (4.4) (38-55)	<0.001*
≤40 (n = 20)	18 (23.4)	2 (6.1)	<0.001*
41-45 (n = 64)	59 (76.6)	5 (15.2)	
46-50 (n = 14)	0 (0)	14 (42.4)	
50-55 (n = 12)	0 (0)	12 (36.4)	
BMI^1^ (kg/m^2^) mean (SD) (range)	28.0 (3.7) (20-37)	29.5 (3.8) (21-40)	0.060
Normal (n = 15)	12 (15.6)	3 (9.1)	0.063
Overweight (n = 47)	37 (48.1)	10 (30.3)	
Obese (n = 48)	28 (36.4)	20 (60.6)	
Smoking status			
Non-smokers (n = 86)	53 (68.8)	33 (100)	<0.001*
Smokers (n = 24)	24 (31.2)	0 (0)	
Nationality^2^			
UAE nationals (n = 19)	12 (15.6)	7 (21.2)	0.416
African (n = 5)	4 (5.2)	1 (3.0)	
Asian (n = 70)	47 (61.0)	23 (69.7)	
Middle Eastern (n = 16)	14 (18.2)	2 (6.1)	
Co-morbidities			
Hypertension (n = 63)	37 (48.1)	26 (78.8)	0.003*
Diabetes mellitus (n = 37)	19 (24.7)	18 (54.5)	0.004*
Dyslipidemia (n = 92)	63 (81.8)	29 (87.9)	0.577
Family history (n = 23)	19 (24.7)	4 (12.1)	0.201
Severity of co-morbidities^3^			
None (n = 6)	6 (7.8)	0 (0)	0.027*
Presence of at most one (n = 29)	25 (32.5)	4 (12.1)	
Presence of at most two (n = 39)	25 (32.5)	14 (42.4)	
Presence of at most three (n = 36)	21 (27.3)	15 (45.5)	

Fifteen patients (13.6%) had a normal BMI (<25), 47 (42.7%) were overweight (BMI 25-29), and 48 (43.6%) were obese (BMI ≥ 30) (Table 1). The mean BMI was slightly higher in women, 29.5 (SD = 3.8), compared to men, 28.0 (SD = 3.7). Obesity was more common among women, with 20 (60.6%) classified as obese compared to 28 (36.4%) men. Although this difference did not reach statistical significance (p = 0.063), it may suggest a potential association that warrants further investigation.

Most participants were non-smokers (n = 86, 78.2%). Smoking was reported exclusively by men (n = 24, 31.2%), with none of the female participants identifying as smokers (p < 0.001).

The ethnic groups included in this study were Africans, Middle Easterners, and Asians from Southeast Asia and South Asia. The African countries were Comoros, Kenya, Tanzania, Mauritius, and Algeria. Patients that were from the Eastern Mediterranean region, also referred to as the Middle Eastern population, were from the UAE, Egypt, Iran, Jordan, Lebanon, and Syria. Additionally, this study included patients from the Asian population, Filipinos, Indians, Nepalis, Pakistanis, and Sri Lankans. Most of the participants were Asians accounting for 70 (64%), reflecting the demographics of Dubai, where South Asians account for more than 50% of the population (Table 1). UAE nationals accounted for only 19 (17.2%). Africans accounted for 5 (4.5%) and 16 (14.5%) Middle Eastern. There was no significant difference in gender distribution across the different nationalities (p = 0.416).

Sixty-three (57.3%) patients had HTN, 37 (33.6%) had DM, 92 (83.6%) had DLP, and 23 (20.9%) had a family history (FH) (Table 1). HTN was more common in women (26 (78.8%)) compared to 37 (48.1%) men (p = 0.003). Similarly, DM was more common in women (18 (54.5%)) compared to 19 (24.7%) men (p = 0.004). DLP and FH were comparable between men and women. Regarding the severity of co-morbidities, none of the patients had all four co-morbidities (HTN, DM, DLP, and FH), and only men had only one co-morbidity (6 (7.8%)) (Table 1). However, women exhibited greater severity of co-morbidities than men (p = 0.027). Women had multiple co-morbidities, with 15 (45.5%) of women having at least three compared to 21 (27.3%) of men (Table 1).

Given that the study was conducted in the UAE, we explored potential differences in demographics and cardiovascular co-morbidities in patients with premature CAD between UAE nationals (19 (17.3%)) and expatriates (non-nationals) (91 (82.7%)) (Table 2). There were no statistically significant differences in demographic variables such as age, gender, BMI, or smoking habits between UAE nationals and non-nationals. Also, co-morbidities (HTN, DLP, and FH) were similar between the two groups. However, a statistically significant difference was observed in the proportion of DM in patients with premature CAD, which was lower in the UAE nationals (2 (10.5%)) compared to the non-nationals (35 (38.5%)) (p = 0.030).

**Table 2:** Demographic characteristics and cardiovascular co-morbidities by UAE nationals UAE: United Arab Emirates; SD: standard deviation; BMI: body mass index ^1^Normal weight BMI (18.5-24.9), overweight BMI (25.0-29.9), and obese BMI (≥30). ^2^Hypertension, diabetes mellitus, dyslipidemia, and family history. *p < 0.05 indicates statistical significance.

Characteristics	UAE nationals (n = 19) N (%)	Non-UAE nationals (n = 91) N (%)	p-value
Gender			
Male (n = 77)	12 (63.2)	65 (71.4)	0.474
Female (n = 33)	7 (36.8)	26 (28.6)	
Age (years) mean (SD) (range)	44.0 (6.3) (29-55)	44.0 (4.6) (27-55)	0.91
≤40 (n = 20)	5 (26.3)	15 (16.5)	0.470
41-45 (n = 64)	8 (42.1)	56 (61.5)	
46-50 (n = 14)	3 (15.8)	11 (12.1)	
50-55 (n = 12)	3 (15.8)	9 (9.9)	
BMI^1^ (kg/m^2^) mean (SD) (range)	29.6 (5.0) (23-40)	28.2 (3.5) (20-35)	0.415
Normal (n = 15)	2 (10.5)	13 (14.3)	0.679
Overweight (n = 47)	7 (36.8)	40 (44.0)	
Obese (n = 48)	10 (52.6)	38 (41.8)	
Smoking status			
Non-smokers (n = 86)	13 (68.4)	73 (80.2)	0.257
Smokers (n = 24)	6 (31.6)	18 (19.8)	
Co-morbidities^2^			
Hypertension (n = 63)	10 (52.6)	53 (58.2)	0.653
Diabetes mellitus (n = 37)	2 (10.5)	35 (38.5)	0.03*
Dyslipidemia (n = 92)	15 (78.9)	77 (84.6)	0.554
Family history (n = 23)	2 (10.5)	21 (23.1)	0.353

As the population predominantly consisted of Asians, a comparative analysis between Asians (70 (63.6%)) and non-Asians (40 (36.4%)) was conducted (Table 3). The results showed that the demographic variables were similar between both groups. However, Asians were found to smoke (10 (14.3%)) significantly less than non-Asians (14 (35.0%)) (p = 0.016). As for co-morbidities, Asians with premature CAD had more DM (32 (45.7%)) compared to non-Asians (5 (12.5%)) (p < 0.001).

**Table 3:** Demographic characteristics and cardiovascular co-morbidities by Asians and non-Asians UAE: United Arab Emirates; SD: standard deviation; BMI: body mass index ^1^Normal weight BMI (18.5-24.9), overweight BMI (25.0-29.9), and obese BMI (≥30). ^2^Hypertension, diabetes mellitus, dyslipidemia, and family history. *p < 0.05 indicates statistical significance.

Characteristics	Asian (n = 70) N (%)	Non-Asian (n = 40) N (%)	p-value
Gender			
Male (n = 77)	47 (67.1)	30 (75.0)	0.517
Female (n = 33)	23 (32.9)	10 (25.0)	
Age (years) mean (SD) (range)	44.3 (4.8) (27-55)	43.6 (5.3) (29-55)	0.469
≤40 (n = 20)	11 (15.7)	9 (22.5)	0.737
41-45 (n = 64)	42 (60.0)	22 (55.0)	
46-50 (n = 14)	10 (14.3)	4 (10.0)	
50-55 (n = 12)	7 (10.0)	5 (12.5)	
BMI^1^ (kg/m^2^) mean (SD) (range)	28.4 (3.5) (21-35)	28.5 (4.3) (20-40)	0.907
Normal (n = 15)	9 (12.9)	6 (15.0)	0.839
Overweight (n = 47)	29 (41.4)	18 (45.0)	
Obese (n = 48)	32 (45.7)	16 (40.0)	
Smoking status			
Non-smokers (n = 86)	60 (85.7)	26 (65.0)	0.016*
Smokers (n = 24)	10 (14.3)	14 (35.0)	
Co-morbidities			
Hypertension (n = 63)	44 (62.9)	19 (47.5)	0.161
Diabetes mellitus (n = 37)	32 (45.7)	5 (12.5)	<0.001*
Dyslipidemia (n = 92)	62 (88.6)	30 (75.0)	0.106
Family history (n = 23)	19 (27.0)	4 (10.0)	0.05*
Severity of co-morbidities^2^			
None (n = 6)	1 (1.4)	5 (12.5)	<0.001*
Presence of at most one (n = 29)	14 (20.0)	15 (37.5)	
Presence of at most two (n = 39)	22 (31.4)	17 (42.5)	
Presence of at most three (n = 36)	33 (47.1)	3 (7.5)	

Also, Asians exhibited a greater burden of co-morbidities compared to non-Asians, with 33 (47.1%) having at least three co-existing risk factors (DM, HTN, DLP, or FH), compared to 3 (7.5%) in non-Asians (p < 0.001). This reflects the number of co-morbidities, not disease severity or control status.

As co-morbidities (HTN, DM, DLP, and FH) are key risk factors for early-onset CVD, we also analyzed the data in relation to BMI (Table 4), age groups (Table 5), and smoking habits (Table 6). HTN was significantly more common among overweight (25 (53.2%)) and obese (35 (72.9%)) patients compared to normal weight subjects (3 (20.0%)) (p = 0.001) (Table 4). This significant increase was observed in men who were overweight (18 (48.6%)) and obese (17 (45.9%)) patients with premature CAD, compared to those with normal weight (2 (5.4%)) (p = 0.038). This trend was also observed in women, where HTN was more common in overweight (7 (26.9%)) and obese (18 (69.2%)) compared to those of normal weight (1 (3.8%)) (p = 0.059) (Table 4).

**Table 4:** Characteristics of cardiovascular co-morbidities by body mass index (BMI) and gender ^1^Normal weight BMI (18.5-24.9), overweight BMI (25.0-29.9), and obese BMI (≥30). ^2^Hypertension, diabetes mellitus, dyslipidemia, and family history. *p < 0.05 indicates statistical significance.

BMI^1^	Normal (n = 15) N (%)	Overweight (n = 47) N (%)	Obese (n = 48) N (%)	p-value
Co-morbidities				
Males				
Hypertension (n = 37)	2 (5.4)	18 (48.6)	17 (45.9)	0.038*
Diabetes mellitus (n = 19)	5 (26.3)	7 (36.8)	7 (36.8)	0.283
Dyslipidemia (n = 63)	9 (14.3)	32 (50.8)	22 (34.9)	0.573
Family history (n = 19)	2 (10.5)	11 (57.9)	6 (31.6)	0.582
Females				
Hypertension (n = 26)	1 (3.8)	7 (26.9)	18 (69.2)	0.059
Diabetes mellitus (n = 18)	2 (11.1)	4 (22.2)	12 (66.7)	0.530
Dyslipidemia (n = 29)	3 (10.3)	9 (31.0)	17 (58.6)	0.737
Family history (n = 4)	1 (25.0)	1 (25.0)	2 (50.0)	0.498
Total				
Hypertension (n = 63)	3 (20.0)	25 (53.2)	35 (72.9)	0.001*
Diabetes mellitus (n = 37)	7 (46.7)	11 (23.4)	19 (39.6)	0.128
Dyslipidemia (n = 92)	12 (80.0)	41 (87.2)	39 (81.3)	0.674
Family history (n = 23)	3 (20.0)	12 (25.0)	8 (16.7)	0.566
Severity of co-morbidities^2^				
None (n = 6)	1 (6.7)	1 (2.1)	4 (8.3)	0.103
Presence of at least one (n = 29)	7 (46.7)	15 (31.9)	7 (14.6)	
Presence of at least two (n = 39)	3 (20.0)	19 (40.4)	17 (35.4)	
Presence of at least three (n = 36)	4 (26.7)	12 (25.5)	20 (41.7)	

**Table 5:** Characteristics of cardiovascular co-morbidities by age group ^1^Hypertension, diabetes mellitus, dyslipidemia, and family history. *p < 0.05 indicates statistical significance.

Age (years)	≤40 (n = 20) N (%)	41-45 (n = 59) N (%)	46-50 (n = 19) N (%)	>50 (n = 12) N (%)	p-value
Co-morbidities					
Hypertension (n = 63)	7 (35.0)	32 (50.0)	13 (92.9)	11 (91.7)	<0.001*
Diabetes mellitus (n = 37)	5 (25.0)	19 (29.7)	8 (57.1)	5 (41.7)	0.177
Dyslipidemia (n = 92)	17 (85.0)	52 (81.3)	13 (92.9)	10 (83.3)	0.762
Family history (n = 23)	6 (30.0)	15 (23.4)	1 (7.1)	1 (8.3)	0.262
Severity of co-morbidities^1^					
None (n = 6)	2 (10.0)	4 (6.3)	0 (0)	0 (0)	0.214
Presence of at least one (n = 29)	6 (30.0)	21 (32.8)	1 (7.1)	1 (8.3)	
Presence of at least two (n = 39)	7 (35.0)	20 (31.3)	5 (35.7)	7 (58.3)	
Presence of at least three (n = 36)	5 (25.0)	19 (29.7)	8 (57.1)	4 (33.3)	

**Table 6:** Characteristics of cardiovascular co-morbidities by smoking status ^1^Hypertension, diabetes mellitus, dyslipidemia, and family history. *p < 0.05 indicates statistical significance.

	Non-smoker (n = 86) N (%)	Smoker (n = 24) N (%)	p-value
Co-morbidities			
Hypertension (n = 63)	55 (64.0)	8 (33.0)	0.007*
Diabetes mellitus (n = 37)	28 (32.6)	9 (37.5)	0.635
Dyslipidemia (n = 92)	74 (86.0)	18 (75.0)	0.196
Family history (n = 23)	19 (22.1)	4 (16.7)	0.563
Severity of co-morbidities^1^			
None (n = 6)	3 (3.5)	3 (12.5)	0.125
Presence of at least one (n = 29)	20 (23.3)	9 (37.5)	
Presence of at least two (n = 39)	33 (38.4)	6 (25.0)	
Presence of at least three (n = 36)	30 (34.9)	6 (25.0)	

Patients with early-onset CVD older than 40 years showed to be more hypertensive compared to those younger than 40 years (Table 5). In the age group 46-50 years, 13 (92.9%) had HTN; among those older than 50 years, 11 (91.7%) were hypertensive compared to the younger age groups (7 (35%)) (p = 0.001). However, there was no significant relationship between age and the other co-morbidities (DM, DLP, and FH).

Also, a significant association between smoking status and HTN was observed, with 55 (64.0%) of non-smokers having HTN compared to only eight (33.0%) of smokers (p = 0.007) (Table 6). Smoking status was not found to be associated with the other co-morbidities (DM, DLP, and FH). As the severity of co-morbidities (HTN, DM, DLP, and FH) an individual has is a major risk factor for early-onset CVD, we analyzed the data in relation to BMI (Table 4), age groups (Table 5), and smoking habits (Table 6). The severity of co-morbidities did not show any significant differences between the BMI categories (Table 4), age groups (Table 5), and smoking habits (Table 6).

## Discussion

Given that the study was conducted in the UAE, a nation characterized by a diverse population with a significant proportion of residents being non-nationals, it was necessary to analyze the data based on nationality and subdivided into ethnic groups. As of 2020, non-Emirati residents accounted for approximately 88% of the UAE's population, with Emirati nationals comprising the remaining 12% [24]. The analysis showed that the representation of nationals and non-nationals in the study mirrored the general population statistics of the country, enabling the researchers to explore potential differences in health outcomes between these groups. Most of the population in the data were South Asians, which was appropriately reflective of the region's demographic as, at a national level, South Asians constituted around 50% of the UAE's total population, making them the largest expatriate group in the country [25]. However, global research indicates that South Asians are the most susceptible to developing CAD due to a combination of factors compared to the general population [26].

Research has shown that South Asians have a higher level of coronary artery calcium, a key marker for atherosclerosis, which contributes to the development of earlier CAD. Additionally, South Asians tend to store body fat in visceral areas rather than subcutaneously, leading to greater metabolic damage and an increased risk of vascular complications [27]. Genetic predisposition also plays an important role, as individuals of South Asian heritage frequently inherit higher lipoprotein levels compared to other ethnic groups making them more susceptible to CVD. The increased prevalence of CAD in South Asians is also attributed to SES and lifestyle factors, particularly diet. Indians, who constitute a large portion of the South Asian population, predominantly follow a vegetarian diet. However, this diet often tends to be high in saturated fats and refined carbohydrates while lacking sufficient omega-3 fatty acids, which are known for their cardioprotection. These findings highlight the urgent need for targeted preventive strategies tailored to the specific characteristics of the South Asian population. Raising awareness about the importance of managing modifiable risk factors and promoting early screening in this high-risk group are essential to reducing the burden of CAD [28].

Our study showed a higher number of expatriate diabetics presented to the emergency department at the tertiary hospital in Dubai with premature CAD. This finding contrasts with the general population demographics, as regional statistics consistently indicate a higher prevalence of diabetes among UAE nationals compared to expatriates. According to the Dubai Household Health Survey (2014-2017), the prevalence of diabetes was 19% among UAE nationals and 14.7% among expatriates [29]. Several studies, including this one, have reported a greater number of diabetic UAE nationals in the general population, which was attributed to multiple factors. Genetic predisposition plays a significant role, with approximately 64% of UAE nationals with diabetes reporting an FH of the condition. Additionally, sedentary lifestyles, unhealthy diets, and high obesity rates are more prevalent among UAE nationals, further contributing to the increased risk of diabetes [30].

Despite regional trends, this study found a statistically significant predictor between expatriates with diabetes presenting with premature CAD compared to UAE nationals with diabetes. This disparity can be attributed to several factors related to socioeconomic conditions, healthcare access, and population demographics. The hospital where the study was conducted is in a low- to middle-income area of Dubai, which serves as the primary home to labor migrants and low-income expatriate workers. Factors such as the availability of low-rent accommodations contribute to the high concentration of expatriates in this region. As a result, most expatriates presenting to the emergency department are predominantly low-income workers who often face significant barriers to adequate healthcare. Many of these individuals have limited access to early screening, preventive care, and chronic disease management services. Research has also found that undiagnosed diabetes is more common among expatriates in the UAE. As a result, their diabetes often remains undiagnosed or poorly managed, increasing their risk of complications such as premature CAD. This lack of proper disease management makes them more likely to experience deterioration and present to the nearest hospital with chest pain due to an episode of premature CAD [31].

In contrast, although UAE nationals have a higher prevalence of diabetes, they often benefit from better healthcare access due to their stronger financial stability, as indicated by multiple studies showing that UAE nationals generally have stronger financial health than expatriates [32]. Additionally, government hospitals provide free healthcare to the local population, further improving access to medical services. As a result, UAE nationals are more likely to receive an earlier diagnosis, better disease management, and preventive interventions, which can delay or prevent severe complications such as premature CAD. These systemic differences suggest that while diabetes is more prevalent among UAE nationals, they are less likely to deteriorate to the point of presenting to the emergency department with premature CAD compared to the low-income expatriate population served by the hospital, which was reflected in this study.

In addition, the study observed a marked rise in HTN diagnoses after the age of 45 within the population. While this trend may partly be attributed to age-related lifestyle factors such as reduced physical activity and associated metabolic changes, it may also reflect delayed diagnosis resulting from infrequent screening and limited access to healthcare services. Undiagnosed HTN in earlier years may go unnoticed until symptoms or complications emerge, often due to the absence of regular medical evaluations.

These findings highlight socioeconomic and healthcare disparities' critical role in health outcomes and underscore the need for targeted public health interventions. Addressing the challenges faced by low-income expatriates while simultaneously catering to the specific needs of each population group is essential. Broader interventions to reduce diabetes-related complications among these low-income populations and the UAE population are necessary.

Timely identification of premature CAD risk factors is important as prevalence is increasing both globally and in the UAE. A study conducted in the region found that the incidence of premature coronary heart disease (CHD) occurs about 10-15 years earlier than that in Western populations [33]. Several studies conducted in the UAE further reflect the increasing prevalence of premature CAD. For instance, a study conducted in Abu Dhabi in the past three years found that nearly half of the admitted heart attack patients were under the age of 50 [34]. The UAE-based studies have also identified the risk factors of HTN, DLP, and obesity to be significantly associated with CAD, further validating the findings of this study. However, while other research has also found that DM and smoking are significantly associated with premature CAD, our study did not observe a significant correlation between these factors. This discrepancy could be attributed to differences in sample size, as the UAE-based study included 20,000 adults, a significantly larger sample size than ours. A larger sample size in our study may have been necessary to establish significant associations with these specific risk factors.

In our study, Emirati national patients diagnosed with premature CAD were mainly overweight, suggesting a significant association between high BMI and the development of CAD in UAE nationals. When comparing the association of DLP, gender, and BMI, the study found that there is no significant association between DLP and either gender or BMI among young adult patients. These findings contribute to a better understanding of the relationship between DLP and gender and BMI in the context of CAD in young adults. Research conducted on young patients in Asia supports these findings that there is no association between DLP and gender [35]. The lack of association between DLP and BMI could also be due to the small sample size. However, it is also possible that patients with DLP are at risk of developing CAD regardless of having a normal or high BMI. This could suggest that DLP is a potentially more dangerous risk factor for CAD than BMI. In contrast, when comparing the associations of HTN and BMI, the results were significant. These findings indicate that having both HTN and high BMI increases the likelihood of developing CAD more than having HTN with a normal BMI.

HTN was also found to be significantly associated with gender. The only other study conducted in the UAE on this association also found significance between HTN and gender; however, while our study found a higher prevalence of HTN in female patients, the previous study reported a higher prevalence in male patients [36]. This could be due to the differences in the study populations. The previous study had a lower proportion of Indian women than this study, as research has shown that Indian women have a higher prevalence of HTN than Indian men [37]. Another factor could be the age range of the participants, as our study focused on younger patients. In contrast, the other study included patients of all ages, potentially influencing the observed gender differences in HTN prevalence.

Most of the patients in our study were male, which is reflective of global research indicating that males have a higher risk of developing CAD than women. Studies have shown that cholesterol is more likely to accumulate in major arteries in men than in women [38]. Additionally, estrogen production in premenopausal women has been found to have protective effects on the cardiovascular system, reducing the risk of atherosclerosis development. Since this research focuses on premature CAD, it is more likely to include premenopausal women with cardioprotective estrogen production, further supporting the observed results [6,7]. These findings highlight the importance of male education about CAD and promoting healthier lifestyle choices to mitigate their elevated risk.

In addition to gender, the study identified other significant risk factors that align with existing academic research. Notably, a substantial proportion of patients presenting with premature CAD at MWEL were also diagnosed with DLP, highlighting the critical importance of lipid profile management in preventing CAD. This finding is consistent with research demonstrating that treating hyperlipidemia reduces cardiovascular mortality [39]. Other significant risk factors observed in the study were the presence of HTN and high BMI, which emphasize the importance of weight loss and blood pressure control in CAD management. In addition, women in this study were more likely to be obese than men, which is reflective of the obesity data in the UAE, according to a study conducted by the UAE Ministry of Health and Prevention (MoHAP), which reported that obesity is higher in women than men in this region [40]. In addition to obesity, certain co-morbidities, such as diabetes, are also more common in women than men, a trend that was reflected in this study, as well as a greater severity of co-morbidities in women. Research shows that the higher prevalence of diabetes in women can be attributed to factors such as body fat distribution, as women generally have higher levels of subcutaneous fat and are more prone to visceral fat accumulation after menopause, which increases insulin resistance and the risk of diabetes. Higher sedentary behavior also contributes to this increased risk [41].

Furthermore, results showed a higher proportion of HTN in non-smokers compared to smokers. The observed trend of higher HTN rates among non-smokers in the study could be attributed to patients quitting smoking after receiving a HTN diagnosis, a phenomenon supported and observed by research indicating that smoking cessation often follows the diagnosis of chronic heart conditions like HTN or CVD, as patients seek to improve their health outcomes. According to the European Respiratory Journal, the CLARIFY registry, involving over 32,000 patients with CAD, revealed that a significant proportion of smokers quit smoking shortly after their diagnosis, with nearly 73% discontinuing within the first year. Smoking cessation during this period substantially reduced cardiovascular risks, suggesting that such behavioral changes are common after diagnosis [42]. Similarly, smoking cessation is recognized as a crucial part of HTN management due to its potential to stabilize blood pressure and improve overall cardiovascular health [43]. This aligns with the findings of low smoking prevalence, as patients may have quit smoking following a diagnosis of HTN or other related co-morbidities.

In this study, known risk factors were assessed in relation to the presence of co-morbidities, and the results indicated that non-smokers were more likely to have severe cardiovascular illness. In contrast, smokers had fewer co-morbidities, with a greater proportion reporting no underlying health conditions compared to non-smokers. This discrepancy might be attributed to individuals quitting smoking after being diagnosed with conditions like HTN, as discussed earlier. Smoking cessation can be triggered by many factors, some of which are often triggered by health concerns and CVD diagnosis.

Patients with a normal BMI had the lowest overall burden of co-morbidities. In contrast, overweight individuals were more likely to present with multiple co-morbid conditions, while obese individuals exhibited the highest co-morbidity burden. These findings align with existing research demonstrating a clear association between increasing BMI and a higher prevalence of CVD and other chronic co-morbidities [44]. Studies have consistently shown that overweight or obese individuals are at greater risk of developing HTN, DLP, type 2 diabetes, and cardiovascular events compared to those with a normal BMI [45]. The relationship between excess adiposity and increased inflammatory processes, insulin resistance, and endothelial dysfunction provides a well-recognized biological pathway linking higher BMI to the development and progression of CVD and multimorbidity [46].

Study limitation

Although a key limitation of this study is its descriptive design, which prevents establishing direct causality between risk factors and premature CAD, longitudinal studies are needed to confirm these associations and identify direct risk factors. However, this study played a crucial role in identifying patterns and highlighting the at-risk population, serving as a foundation for future research and targeted interventions.

Additionally, co-morbidity severity was not uniformly captured, limiting our ability to assess each patient's true disease burden. The exclusive use of data from a CATH LAB may also introduce selection bias, as only patients with more advanced or symptomatic diseases are typically referred for catheterization.

Furthermore, while all smokers in our study were men, the gender disparity in smoking behavior should be further explored for its potential contribution to cardiovascular risk factors. The smoking data was also limited by incomplete documentation of pack-years in many patients. Since most individuals presented through the emergency department, detailed histories were sometimes difficult to obtain, which may have led to underreporting or missing information on relevant risk factors.

Additionally, the relatively small sample size and single-center study design may limit the generalizability of the findings to the broader UAE population. Lastly, the study did not account for potential confounding variables such as occupation, dietary patterns, or medication use, all of which may significantly influence cardiovascular risk. Future studies should aim to incorporate these elements to provide a more comprehensive understanding of premature CAD in diverse populations.

## Conclusions

The primary aim of our study was to assess the major cardiovascular determinants associated with the development of premature CAD within a tertiary care hospital in Dubai. A thorough analysis of medical records suggested an association between CAD and individuals of South Asian descent, with South Asian men being the most affected in comparison to the general population. Additionally, HTN, DLP, and elevated BMI (overweight and obese) were identified as significant contributors to disease progression. Data also showed that the disease was most prevalent among Asians, specifically, South Asian men. These finding highlights the need for specialized preventive care and targeted interventions for high-risk populations.

This research provides valuable insights into investigating the major determinants of early-onset CAD in Dubai, UAE. The findings hold significant implications for healthcare professionals and policymakers, advocating for a targeted approach to CAD prevention, especially among South Asian communities. Preventive strategies should prioritize the management of DLP and HTN, along with effective weight control interventions, to help reduce the regional burden of premature CAD. Early screening programs, lifestyle modifications, and culturally tailored healthcare initiatives could improve cardiovascular outcomes and lower the incidence of early-onset CAD in high-risk populations.
